# Early versus Delayed Antiretroviral Therapy for HIV and Tuberculosis Co-Infected Patients: A Systematic Review and Meta-Analysis of Randomized Controlled Trials

**DOI:** 10.1371/journal.pone.0127645

**Published:** 2015-05-22

**Authors:** Shipeng Yan, Lizhang Chen, Wenqiong Wu, Zhongxi Fu, Heng Zhang, Zhanzhan Li, Chenchao Fu, Jingsong Mou, Jing Xue, Yingyun Hu

**Affiliations:** 1 The Affiliated cancer hospital of Xiangya Scholl of Medicine, Central South University, Changsha, 410013 China; 2 Department of Epidemiology and Health Statistics, School of Public Health, Central South University, Changsha, Hunan Province, 410078 China; 3 Centers for Disease Control and Prevention of Hunan Province, Changsha, Hunan Province, 410005 China; 4 Centers for Disease Control and Prevention of Changsha City, Changsha, Hunan Province, 410013 China; 5 Xiangya Hospital, Central South University, Changsha, Hunan Province, 41008 China; 6 Changsha Medical University, Changsha, Hunan Province, 410000 China; Imperial College London, UNITED KINGDOM

## Abstract

**Objective:**

To compare important clinical outcomes between early and delayed initiation of antiretroviral therapy (ART) in adults who had a co-infection of human immunodeficiency virus (HIV) and tuberculosis (TB).

**Methods:**

We performed a systematic search for relevant publications on PubMed, EMBASE, and the International Clinical Trials Registry Platform. We included randomized controlled trials (RCTs) that compared early ART initiation (within four weeks after anti-TB treatment starting) and delayed ART initiation (after eight weeks but less than twelve weeks of anti-TB treatment starting) in the course of TB treatment. Pooled estimates with corresponding 95% confidence interval (95%CI) were calculated with random-effects model. Sensitivity analysis was performed to investigate the stability of pooled estimates.

**Results:**

A meta-analysis was evaluated from six RCTs with 2272 participants. Compared to delayed ART initiation, early ART initiation significantly reduces all-cause mortality in HIV-positive patients with TB [incidence rate ratio (IRR) 0.75, 95%CI 0.59 to 0.95; I^2^ = 0.00%; p = 0.67], even though there is an increased risk for IRD [IRR 2.29, 95%CI 1.81 to 2.91; I^2^2 = 0.00%; p = 0.56]. Additionally, early ART initiation was not associated with an increased risk for grade 3-4 drug-related adverse events [IRR 0.99, 95%CI 0.83 to 1.18; I^2^ = 0.00%; p = 0.56].

**Conclusions:**

Although limited evidence, our results provide support for early ART initiation in the course of anti-TB treatment. However, more well-designed cohort or intervention studies are required to further confirm our findings.

## Introduction

Co-infection with human immunodeficiency virus (HIV) and tuberculosis (TB) poses one of the major ongoing challenge for global tuberculosis (TB) and acquired immune deficiency syndrome (AIDS) prevention and control [1, 2]. HIV significantly increases individual risk of progression to active TB in both primary TB and the reactivation of latent TB. Likewise, TB is associated with worsening of the immune suppression, which partly results in a high incidence of death and opportunistic infection [3, 4, 5]. It is estimated that among 33.4 million people living with HIV worldwide, about one-third are also infected by MTB [6]. In 2012, of the estimated 1.2 million TB deaths, about one-quarter were HIV/TB co-infected patients [7]. While the expanding access to antiretroviral therapy (ART) dramatically decreased the morbidity and mortality of HIV/TB co-infection, individuals with HIV infection still suffered from an excess risk of 10-fold for TB development and a higher TB-associated death rate than those HIV-uninfected individuals [3, 8].

As an important part of improving outcomes of HIV/TB care, over the years, to optimize the treatment strategies among patients of HIV/TB co-infection has aroused a lot of concerns, of which the most essential one is to find an optimal timing to initiate ART. Observational studies and randomized controlled trials (RCTs) have revealed that different timings of ART initiation during TB treatment could lead to distinct clinical outcomes [9, 10, 11, 12]. However, although the majority of these studies suggested that early ART initiation was associated with a better survival, the findings are controversial. These conflicting results may lead to a considerable ambiguity and doubt in patients and healthcare providers when facing treatment decisions for HIV/TB co-infection. Therefore, unveiling the underlying pattern of the association between timing of ART initiation and clinical outcomes by summarizing the available evidence is important for making evidence-based recommendations.

Long recognized as an important statistical technique for the integration of known research evidence, meta-analysis has been applied to evaluate the threshold conditions of ART initiation in HIV-associated opportunistic infection. For instance, based upon a meta-analysis of two RCTs, Njei et al. found that there was no significant difference in the risk of mortality between early and delayed initiation of ART in HIV-positive patients with concurrent cryptococcal meningitis (CM), and suggested that practitioners and policy-makers may consider delaying initiating ART for HIV patients who present to health services and are diagnosed with CM because of the possible increase in mortality associated with immune reconstitution disease (IRD) [13]. Although previous studies [14, 15, 16] made several attempts to sum up the evidence on the optimal timing of ART initiation in the course of TB treatment, as far as we known, no meta-analysis have ever been conducted. Therefore, to accumulate and update evidence for the management of ART in HIV/TB co-infected patients, we carried out this meta-analysis to compare the important clinical outcomes of early versus delayed ART initiation.

## Methods

This systematic review was conducted in line with the guidelines of CHSRI (Cochrane Handbook for Systematic Reviews of Interventions) [17] and in accordance with a priori protocol agreed by all authors.

### Search strategies

A systematical computer search of the publications was performed in PubMed, Embase, and the International Clinical Trials Registry Platform, using a combination of the search terms: “antiretroviral therapy”, “highly active anti-retroviral therapy”, “HAART”, “tuberculosis”, “TB”, “human immunodeficiency virus”, “HIV”, “acquired immune deficiency syndrome”, “AIDS”, and “HIV/TB”. The search was limited to human subjects and RCTs. No language, publication, or date restrictions were imposed in July 2014. Manual searches of reference lists and journals were conducted for other potentially eligible studies. Authors of studies were contacted for clarifications and additional information when necessary.

### Inclusion and exclusion criteria

According to the recommendations from the CHSRI, eligibility criteria were based on key study features: design, participants, comparator, outcome, and length of follow-up [17]. Specifically, trials were eligible for inclusion in the current study if they met the following criteria: (1) study design: RCT; (2) population: HIV-positive adults (>13y) who have a co-infection with probable or confirmed TB; (3) intervention: patients were assigned to either an early ART initiation group or a delayed ART initiation group, regardless of any combinations of ART and anti-TB drugs used. We defined early treatment group as an ART initiated within four weeks after anti-TB treatment starting, whereas delayed treatment group was defined as an ART initiated after eight weeks but less than twelve weeks of anti-TB treatment starting; (4) studies should contain data for at least one of the following outcomes: mortality, drug-related adverse events, and IRD; and (5) the participants were followed-up at least 6 months (since some of adverse events may be time-dependent, such as IRD).

Studies were excluded for following reasons: (1) considering that there is obviously clinical heterogeneity between patients with TB meningitis and patients with extra-cranial TB, we specifically excluded studies of TB meningitis; (2) to enhance the comparability of intervention, we excluded studies that did not compare the timing of ART initiation in the course of anti-TB treatment.

### Study selection and data extraction

The search results were imported into bibliographic citation management software (EndNote X7) and excluded duplicate references. S.P.Y., W.Q.W. and Z.Z.L. independently reviewed and selected all potential available articles by checking the title and abstract. If articles were considered to be potential eligible for final analysis, the full-text of studies was evaluated. Any disagreement among reviewers was resolved by reappraised the original literatures and discussing the objections with the fourth one author (L.Z.C.). Two authors (Z.X.F. and H.Z.) performed data extraction procedure using a standardized questionnaire. Extracted data included general information (title, first author, year of publication, journal and study setting), study characteristics (design, follow-up, inclusions/exclusions), participants characteristics (mean age, the proportion of male, baseline median CD4 cell count and HIV load), and results, i.e. number of events by comparison group, hazard ratio (HR), incidence rate ratio (IRR) as reported, and any other raw data for effect calculation.

### Assessment of quality of included studies

The Cochrane Collaboration's risk of bias (ROB) tool was used to evaluate the methodological quality and risk of bias in studies meeting eligibility criteria from following 6 domains: sequence generation, allocation concealment, blinding, incomplete outcome data, selective outcome reporting, and other potential biases. In addition, we assessed the quality of evidence of each outcome, as opposed to individual studies, using the GRADE (Grades of Recommendation Assessment, Development and Evaluation) methods. The GRADE methods ranks quality of evidence into four grades: high, moderate, low and very low. Evidence from RCTs was rated as the highest levels at first, but can downgrade into other levels, depending on its study limitations, indirectness of evidence, heterogeneity and imprecision of results, as well as the probability of publication bias[18].

### Date synthesis and statistical analysis

We used the reported effect estimates if provided in study reports, but when necessary, we calculated the effect estimates (express as incidence rate ratio [IRR]) and its 95% confidence interval (95%CI) with Newcombe-Wilson method [19]. The reported or calculated effect estimates across studies was pooled to estimate the overall effect. Because of anticipative clinical and methodological heterogeneity of the studies, we pooled data with the random effects model of DerSimonian and Laird random. Heterogeneity between studies was assessed using the I^2^ statistic. I^2^ value ≤ 25% indicated lower heterogeneity, I^2^ value >25% but ≤75% indicated moderate heterogeneity, I^2^ value >75% indicated higher heterogeneity [20]. In order to explore the potential heterogeneity, we planned to performed several subgroup analyses if the enough data were provided. These planned subgroup analysis will based on CD4 count stratification (CD4 count <50 or >50 cells/mm^3^), site of TB disease (intracranial and extra-cranial), and the modality of diagnosis (confirmed or probable TB).

Publication bias was evaluated by funnel plot or Egger’s tests. If there is evidence of publication bias, we applied a nonparametric trim-and-fill method to adjust the results of meta-analysis [21]. Additionally, to assess the stability of our primary outcomes, a sensitivity analysis was conducted basing on the different statistical model and quality of studies. All of the data sorting and statistical analysis was undertaken by J.S.M and C.C.F. using the Stata statistical software (Version 11.0, Stata Corp, College Station, TX).

## Results

### Results of the search strategy

**Fig 1:**
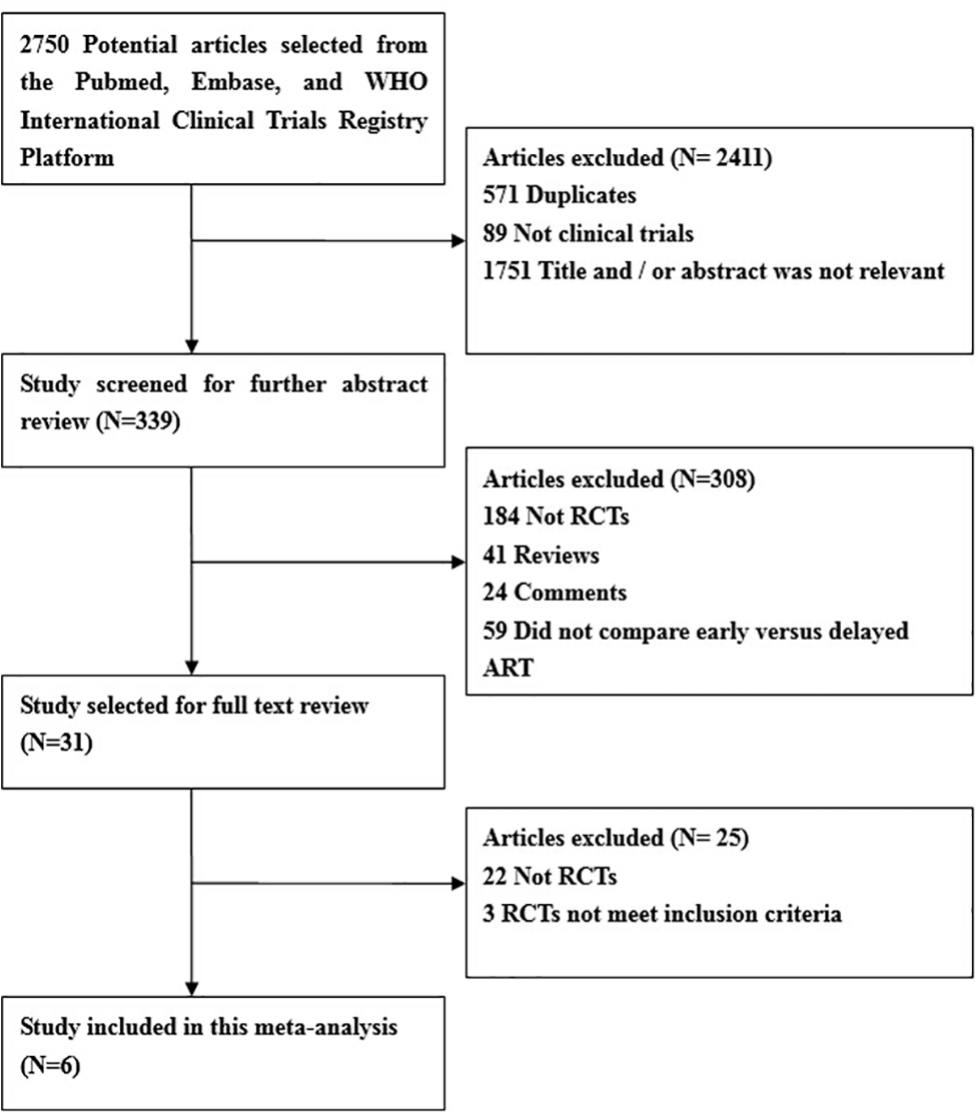
Flow chart of articles identified, screened, assessed and included.

A total of 2750 potential publications were retrieved from the initial database search; of them, 2719 were excluded as duplicated or irrelevant on the basis of the title and abstract. Thus, the full text of the remaining thirty-one studies were obtained and assessed according to the eligibility criteria. Among them, twenty-five studies were excluded because twenty-two were not RCTs, two did not compare the early versus delayed ART initiation in the course of anti-TB treatment [9, 22], and one exclusively focused on patients with tuberculous meningitis [12]. Consequently, six RCTs were ultimately included in this meta-analysis [10, 11, 23, 24, 25, 26]. The flow diagram of the detailed process of study selection is shown in Fig 1.

### Characteristics of included studies

An outline of the six trials was summarized in Table 1. The selected trials included a total sample size of 2272 participants (1153 for early group and 1119 for delayed group), three of which were conducted in South-East Asia[10,24,26], two in Africa[11,23], and one study in a combination of regions in Africa, America and Asia[25]; One study compared clinical outcomes for total lymphocyte count less than 1200 cells/mm^3^[11], four compared clinical outcomes for baseline CD4 counts less than 200 to 500 cells/mm^3^[23,24,25,26], and one compared clinical outcomes with no limitation in baseline CD4 counts[10]; Three studies[11,23,24] reported outcomes which were limited to confirmed (positive smear or culture result) TB cases and the other three [10,25,26] included confirmed and probable TB cases; Drug susceptibility data were reported in four studies[10,23,24,25]. One study [10] only recruited drug-sensitive TB cases and in the remaining studies[23,24,25], the proportion with multi-drug resistance ranged from 2.2% and 4.4%; In addition, four studies[10,23,24,26] reported on TB type: among them, the percentage of individuals with extra-pulmonary TB ranged from 4% to 53%.

**Table 1:** Summary Characteristics of the included studies.

	Shao (2009)[11]	Abdool Karim (2011)[23]	Blanc (2011)[24]	Havlir (2011)[25]	Manosuthi (2012)[26]	Sinha (2012)[10]
Location of study	Tanzania	South Africa	Cambodia	Africa,Asia,America	Thailand	India
Trial design	Open label,RCT	Open label,RCT	Open label, RCT	Open label,RCT	Open label,RCT	Open label,RCT
Main inclusion criteria	Only patients with confirmed TB and total lymphocyte count <1200/mm^3^	Only patients with confirmed TB and baseline CD4 count <500/mm^3^	Only patients with confirmed TB and baseline CD4 count <200/mm^3^	Patients with confirmed or probable TB and baseline CD4 count <250/mm^3^	Patients with confirmed or probable TB and baseline CD4 count <350/mm^3^	Patients with confirmed or probable TB and no restriction with baseline CD4 count
Proportion of extra-pulmonary TB (%)	No reported	4.4	15.9	No reported	52.9	38.0
Proportion of multi-drug resistant TB (%)	No reported	4.4	2.2	4.2	No reported	Only involved drug-sensitive cases
Sample size (early ART vs. delayed ART)	70 (35 vs. 35)	429 (214 vs. 215)	661(332 vs. 329)	806 (405 vs. 401)	156 (79 vs. 77)	150 (88 vs. 62)
Timing of ART initiation relative to the anti-TB treatment						
Early ART initiation	2 weeks	4 weeks	2 weeks	2 weeks	4 weeks	2~4 weeks
Delayed ART initiation	8 weeks	8~12 weeks	8 weeks	8~12 weeks	12 weeks	8~12 weeks
Characteristics of participants						
Mean age (year)	36.2	34.4	35.5	34.0	38.0	34.8
Median CD4 (cells/mm^3^)	104	150	25	77	43	133
Log VL (copies/ml)	No reported	5.2	5.6	5.4	5.7	5.3
Outcomes						
All-cause mortality						
Early ART initiation	5.7%(2/35)	7.0%(15/214)	17.8%(59/332)	7.7%(31/405)	7.6%(6/79)	10.2%(9/88)
Delayed ART initiation	2.9%(1/35)	6.9%(15/215)	27.4%(90/329)	9.2%(37/401)	6.5%(5/77)	11.3%(7/62)
Incidence of IRD events						
Early ART initiation	0.0%(0/35)	20.1% (43/214)	33.1%(110/332)	10.6% (43/405)	32.9%(26/79)	10.2%(9/88)
Delayed ART initiation	0.0%(0/35)	8.4%(18/215)	13.7%(45/329)	4.7% (19/401)	19.5%(15/77)	9.7%(6/62)
Incidence of grade 3–4 drug-related adverse events						
Early ART initiation	14.3%(5/35)	52.3%(112/214)	75.6%(251/332)	43.7%(177/405)	24.0%(19/79)	23.9%(21/88)
Delayed ART initiation	2.9%(1/35)	49.8%(107/215)	74.5%(245/329)	47.4%(190/401)	24.7%(19/77)	22.6%(14/62)
Median follow-up (months)	24	17.7	25	11	12	12

S1 Fig displayed a graphical explanation of the risk of bias across the studies using the tool developed by the Cochrane Collaboration. All of the studies adequately described how the randomized allocation sequence was generated, and all but one [10] of the studies fully concealed the allocation prior to assignment. There are no studies suffered from the influence of attrition bias generated from incomplete outcome data, or suffered from the influence of reporting bias generated from selective outcome reporting. In addition, although all studies are open-label, given the objectivity of clinical outcomes, the performance bias and detection bias appeared to be limited.

### Outcome measures

#### Mortality

**Fig 2:**
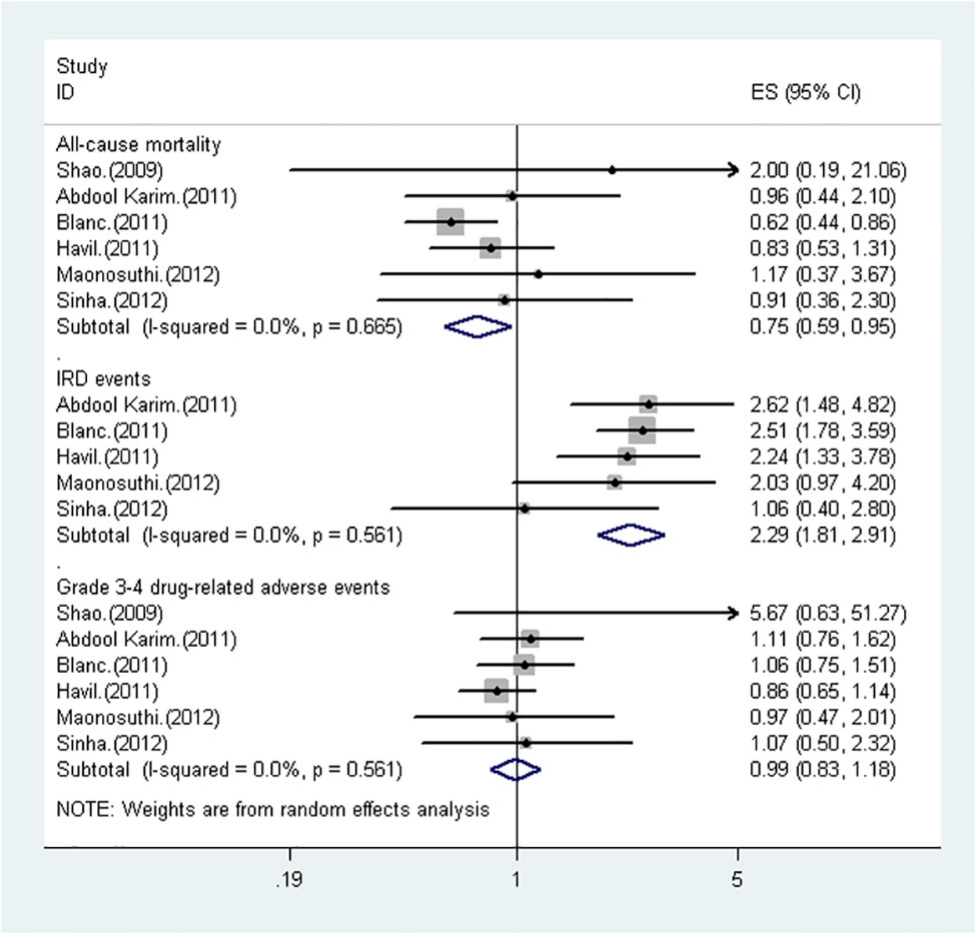
Forest plot of randomized controlled trials comparing the clinical outcomes of early versus delayed ART initiation.

Data on mortality were available from all the included six trials. In group of early ART initiation and group of delayed ART initiation, 1153 and 1119 patients were evaluable for mortality, respectively. Overall, there were 122 deaths in the early ART initiation group and 155 deaths in the delayed ART initiation group. As shown in Fig 2, early ART initiation is associated with a significant reduction in all-cause mortality (pooled IRR = 0.75, 95%CI: 0.59 to 0.95). No statistical heterogeneity was observed (I^2^ = 0.00%, p = 0.67). The quality of evidence from studies for all-cause mortality was low and downgraded due to inconsistence of results and serious imprecision owing to the small number of events.

#### IRD events

Data on IRD events were reported in five trials. 231 of 1118 patients(20.66%)in the early ART initiation group and 103 of 1084 patients(9.50%)in the delayed ART initiation group developed IRD. As presented in Fig 2, early ART initiation was associated with 2.29 times risk of IRD development (95%CI 1.81 to 2.91) compared with delayed ART initiation. No statistical heterogeneity was observed (I^2^ = 0.00%, p = 0.56).The quality of evidence of literatures for this pooled result was ranked as moderate because of very few events.

### Grade 3–4 drug-related adverse events

Data on drug-related adverse events were reported in six trials.585 of 1153 patients(50.74%)in the early ART initiation group and 576 of 1119 patients(51.47%)in the delayed ART initiation group occurred grade 3–4 drug-related events. As shown in Fig 2, no statistical heterogeneity was observed (I^2^ = 0.00%, p = 0.56), and there was no significant difference in drug-related events between the two groups (pooled IRR = 0.99, 95%CI = 0.83 to 1.18). The quality of evidence from studies related to adverse events was high with no observed study limitations.

### Sensitive analysis and Publication bias

**Fig 3:**
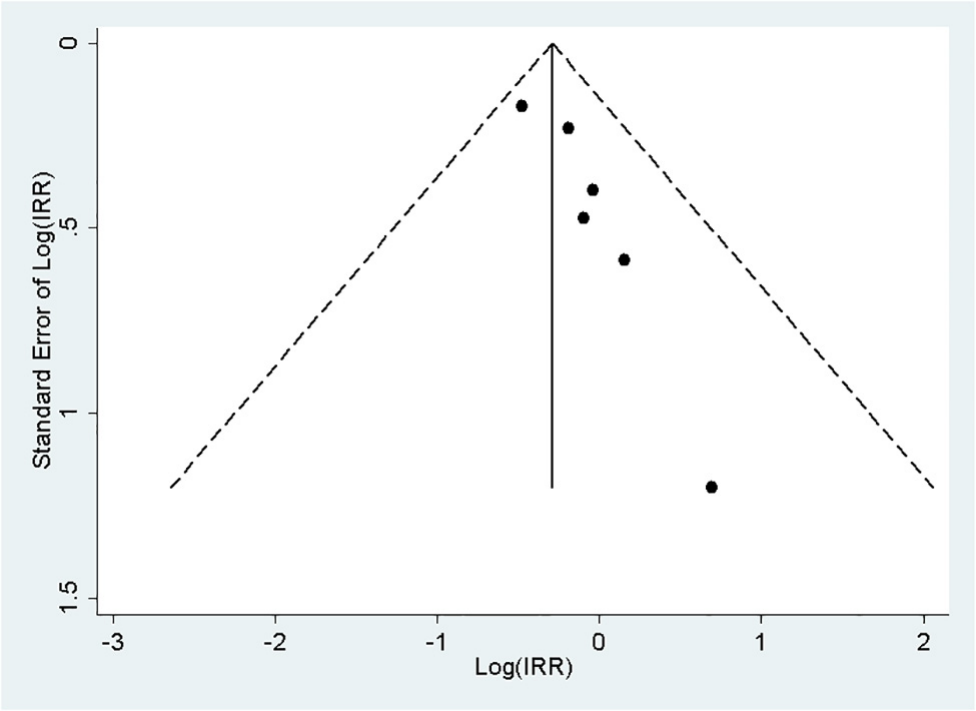
Funnel plot of the publication bias.

We performed sensitivity analyses to evaluate the stability of our pooled analysis. Exclusion of the study which is lack of allocation concealment [10], the pooled results did not change significantly (IRR for mortality: 0.76; IRR for IRD: 2.39; IRR for adverse events: 0.95). Likewise, the sensitivity analysis based on the different statistical model (fix-effect model) did not alter the results of our pooled analysis (IRR for mortality: 0.76; IRR for IRD: 2.11; IRR for adverse events: 0.96).Using all-cause mortality as an endpoint, the funnel plot (Fig 3) and Egger’s test (p = 0.02) suggest the presence of publication bias. To adjust the bias, the trim and fill analysis identified 3 imputed studies and generated an adjusted estimate of 0.70(95% CI: 0.56 to 0.88).

## Discussion

To our knowledge, this is the first meta-analysis evaluating the clinical outcomes of early versus delayed ART initiation in HIV/TB co-infected patients. Our current findings provided evidence, albeit of differing quality depending on the outcome in question, that ART initiation (within four weeks of anti-TB treatment starting) has a significant benefit to avert death, even if there is an increased risk for IRD. We also found that early ART initiation did not increase the risk of grade 3–4 drug-related adverse events. Additionally, although not a specific outcome was reviewed, early ART initiation may be associated with lower likelihood of HIV disease progression [10].

The best timing to initiate ART in patients with HIV-associated TB has been a subject of intense debate. Concern about early ART initiation included a high pill burden, overlapping toxicities and IRD. Conversely, delayed ART initiation may be associated with an increased risk of the AIDS-related illness and death [27, 28]. To evaluate the timing for ART initiation, previous observational studies [29, 30, 31] and a randomized trial conducted by Abdool Karim et al (2010) [22] have shown that concurrent ART significantly improves survival. In 2011, three RCTs provided further evidence to support an early ART initiation (two to four weeks into TB therapy) in the course of anti-TB treatment, especially in patients with profound immunosuppression. In response to these evidences, 2012 WHO guideline recommended that ART should be initiated as soon as possible within the first eight weeks of anti-TB treatment [32]. However, more recently, results from two RCTs conducted in Thailand [26] and India [10] failed to show a significantly survival benefit for patients who initiated ART early, which posed a potential challenge to current WHO recommendations. Thus, to update the evidence base for ART management among HIV/TB co-infected patients, we conducted this systematic review to evaluate the effect of early versus delayed ART initiation. Our results indicated that early ART initiation significantly decreased the all-cause mortality and was not associated with an increased risk for grade 3–4 drug-related adverse events. Sensitivity analyses based on different criteria did not significantly change the pooled results and no statistical heterogeneity was observed among studies. These results may add new evidences to support current WHO recommendations to initiate ART early in the course of anti-TB treatment.

The findings of this meta-analysis must be interpreted cautiously in view of the strengths and limitations of the included trials. A key strength of this study is the fact that all studies included in this meta-analysis were well-performed and high-quality RCTs. Additionally, with the enlarged size of the participants, we have enhanced the statistical power to provide more precise and reliable effect estimates. Nevertheless, despite these advantages, several limitations of the current study should not be overlooked. First, obvious bias in study setting was found. Almost all of studies included in this systematic review come from South-East Asia and Africa, with little contribution from Europe, North America and Australia, which may limit the generalizability of our conclusions to resource-rich environments. Secondly, the proportion of patients with known drug-resistant TB was generally lower in this meta-analysis, thus results here may not be applicable to this population. Additionally, because we specially excluded studies on TB meningitis, the conclusions of the meta-analysis cannot be extended to individuals on TB meningitis. In fact, a recent randomized study of adults exclusively on TB meningitis in Vietnam reported extremely high mortality rates in both groups and no benefit of immediate versus early ART[12]. These exceptions withstanding, our findings provide evidence that it is safe to initiate ART within four weeks of anti-TB treatment start, and that it significantly reduces mortality. Third, as with most of meta-analyses, the results of this study are subject to limitations inherent based on pooling data from different trials with different baseline characteristics of randomized patients, lengths of follow-up period, and modality of diagnosis. Although we acknowledge these heterogeneities by reporting results from random effects estimates, these differences between studies may still have a potential impact on our results. Fourth, detailed data within CD4 counts stratification were absent from most studies, making it difficult to assess the effect of early versus delayed ART by the degree of immunosuppression. Results from two trials [23, 25] showed that early ART initiation was associated with a significant survival benefit only in patients with baseline CD4 cell count <50 cells/mm^3^. Current guidelines recommend that ART should be started as a matter of emergency (within 2 weeks after the onset of anti-TB treatment) in TB patients with a CD4 count less than 50 cells/mm^3^ [32]. However, we believe that the issue regarding baseline CD4 counts less than 50 cells/mm^3^ as an indicator of early versus delayed ART initiation is still a matter for argument. Although no detail data were provided, the CAMELIA trail indicated that the benefit of earlier ART did not differ significantly between patients with CD4 cell count <50 cells/mm^3^ and those with higher counts [24]. Fifth, although we have planed to perform a subgroup analysis according to the modality of diagnosis, the paucity of data prevented us to categorize patients to do such a separate comparison. Nevertheless, results from the STRIDE trial showed that there is no difference in mortality between early and delayed ART initiation when stratified by smear-positive and smear-negative TB [25]. Finally, it should be noted that half of the include trails were relative small, and the finding of significant funnel-plot asymmetry may indicate the existence of small-study effects. Thus, there may be a tendency for these small trials to overestimate effects of treatment. In addition, the funnel-plot and Egger’s test suggested there was a significant publication bias in our pooled analysis. Publication bias would still be an issue of concern even if Egger’s test revealed no evidence. The trim and fill method suggests the existence of unpublished hidden studies and yields unbiased pooled estimates. The pooled results changed slightly after adjustment for publication bias, but the significance of the results remained.

In summary, despite the potential limitations of the performed meta-analysis, our findings contributed to the evidence base for the management of ART in HIV/TB co-infected patients. Based on the imperfect published evidence available, our results provide support for early ART initiation in the course of anti-TB treatment. However, the results should be interpreted cautiously in view of the limitations in this study, and the findings must be further clarified through more well-designed cohort or intervention studies.

## Supporting Information

S1 PRISMA Checklist (DOC)

S1 Fig Risk of bias assessment for included trials by the Cochrane risk of bias tool. (DOC)
