# Supplementary material for: Early versus Delayed Antiretroviral Therapy for HIV and Tuberculosis Co-Infected Patients: A Systematic Review and Meta-Analysis of Randomized Controlled Trials
Source: PLoS One. 2015 May 22;10(5):e0127645. doi: 10.1371/journal.pone.0127645 (PMC4441463; doi:10.1371/journal.pone.0127645)
Supplement: S1 Fig — (DOC) [file pone.0127645.s002.doc]

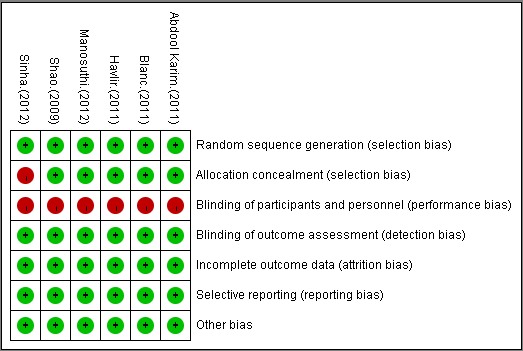


**Figure 1. Risk of bias summary: the authors’ judgments about each risk of bias item for the included studies.**


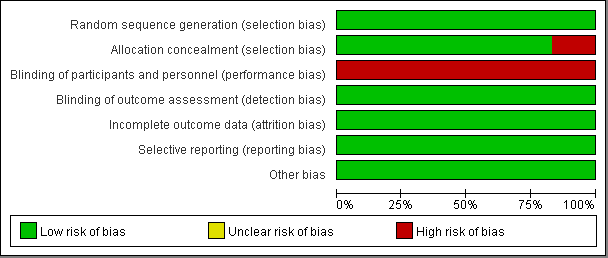


**Figure 2. Risk of bias graph: the authors’ judgments about each risk of bias item presented as percentages across all included studies.**
